# Selective Laser Melting of Molybdenum Alloy on Silicon Carbide Substrate

**DOI:** 10.3390/ma18092121

**Published:** 2025-05-05

**Authors:** Marina Aghayan, Tsovinar Ghaltaghchyan

**Affiliations:** A.B. Nalbandyan Institute of Chemical Physics NAS RA, Paruyr Sevak 5/2, Yerevan 0014, Armenia; tsovinar.ghaltaghchyan@gmail.com

**Keywords:** multilayer manufacturing, silicon carbide, molybdenum silicide, molybdenum

## Abstract

Additive manufacturing (AM) technologies allow for the creation of components with greater design flexibility. The complexity in geometry and composition can enhance functionality, while parts made from multiple materials have the capacity to deliver improved performance. Nonetheless, most multimaterial printing methods are still in their infancy and face numerous challenges. Numerous materials require individual post-treatment, and some may not be compatible with each other regarding shrinkage, melting or sintering temperatures, and interactions. In this study, we introduce a technique for producing a metal–ceramic multimaterial prototype for electronic packages through powder-bed additive manufacturing technology. Silicon carbide-based ceramic substrate was manufactured by selective laser melting, on which molybdenum-based conductive tracks were printed. The results indicated that the SiC-based samples exhibit a relatively uniform microstructure with homogeneously distributed porosity. Mo-based powder containing 5% silicon was successfully SLM-ed on the SiC layer. The microstructural and chemical analyses show that Mo reacted with Si during selective laser melting, resulting in formation of molybdenum silicides. The surface of Mo-based layer surface is smooth; however, there are few cracks on it. The Vickers hardness was measured to be 7.6 ± 1 GPa. The electrical resistivity of the conductive track is 2.8 × 10^−5^ Ω·m.

## 1. Introduction

Molybdenum is a refractory metal which possesses a high melting point (2623 °C) [1], high thermal conductivity (139 W/mK) [1], low electrical resistivity (5.46 × 10^−8^ Ω·m) [2], small thermal expansion coefficient (4.8 × 10^−6^/K) [3], and large elastic modulus at room temperature. Such a combination of properties makes molybdenum a useful material for electronic packages. The thermal conductivity and low specific heat enable Mo to be heated and cooled quickly, making it particularly beneficial in electrical applications. The thermal expansion coefficient is similar to that of silicon and borosilicate glass at cryogenic temperatures, which renders molybdenum a suitable option for space electronics applications [4]. Molybdenum possesses one of the highest melting points among other elements, yet it is prone to oxidation at elevated temperatures. The poor oxidation resistance of pure Mo can be improved when adding such elements as silicon or boron [5].

Oxidation of Mo is repeatedly mentioned as a limiting factor in some applications, or at least a consideration, because mass loss due to oxidation in Mo and its alloys reduces strength compared to an intact specimen. Another limitation in application is the cost of molybdenum.

Additive Manufacturing can produce intricate structural designs with improved functionality and reduced waste. This may lower the expense of the components and broaden the application. Despite the high melting temperature, molybdenum is possible to manufacture by selective laser melting (SLM) technology. There are attempts to manufacture Mo by SLM [6,7,8,9,10,11]. Faidel et al. studied the influence of layer thickness, scanning velocity, and overlap on the density and microstructure of the Mo [12]. They achieved 82.5% density by applying laser power of 200 W. To enhance density, it was advised to utilize increased laser power and reduce layer thickness. Kaserer et al. [13] solved the problem with crack formation by adding 0.45 wt% carbon to molybdenum, which reveals that carbon changes solidification mode, resulting in a larger grain boundary area while minimizing segregated oxygen. Carbon reacts with leftover oxygen in the build chamber and CO eliminates, which is the reason for the lower C and oxygen levels in the final material. The influence of carbon addition to molybdenum also improved the mechanical properties (Table 1). The substrate temperature was necessary to eliminate the cracks and achieve full density. Wang et al. [14] consider that the crack formation during the SLM can be prevented by applying layer-wise scanning rotation. Moreover, high laser power and a tiny structure of supports were proposed to slow the heat transfer and achieve crack-less microstructure of dense molybdenum without adding carbon. The supports allowed the printed components to withstand elevated temperatures for an extended period during heating by providing a low rate of heat elimination.

Higashi [15] investigated the influence of process parameters on the defect formation, focusing on porosity, crystallographic texture, and the characteristics of the melt pool during SLM of Mo. Over 99% theoretical density was achieved by applying > 150 J/mm^3^ volumetric energy density (VED). The influence of VED on the porosity was especially significant at higher layer thickness. It was found that scan speed had a direct impact on the properties of the crystallographic texture.

Kinkade evaluated the influence of energy density, scanning techniques, and environments on the mechanical properties of Mo and Mo alloyed with Rhenium [16]. Similar to other research, Kinkade acknowledged that increased VED resulted in greater densities and improved mechanical characteristics. Eckley et al. [17] revealed that the flexural strength of the printed Mo and Mo-Re alloys enhances when SLM is performed in an argon-3% hydrogen gas environment. However, they exhibited a large degree of anisotropy in mechanical properties that is affected by the build direction. The mechanical properties significantly decreased with increasing scanning speed when printed in the argon-3% hydrogen environment, while there is minimal sensitivity of scanning speed to flexural strength when manufacturing in an argon environment. Conversely, Bustin concluded that the change in H2 amount within the build atmosphere generated only minor effects on the outcomes when compared to laser speed and test temperature [18]. The author suggests using a sufficiently high VED to completely melt the material and minimize porosity, while also maintaining a low laser speed. Table 1 concludes the results of manufacturing Mo by SLM technology.

**Table 1:** Literature review of SLM of molybdenum and its parameters.

Reference	Feedstock Composition	SLM Parameters	Achieved Density	Other Properties
[12]	Mo	Spot velocity—556 mm/s Layer thickness—25 µm Overlap—20 µm Laser power—200 W Energy input—480 J/mm^3^	82.5%	Heat conductivity—142 W/mK (at 20 °C) and 105 W/mK (at 1000 °C) Young’s modulus—330 GPa (at 20 °C) and 280 (at 800 °C)
[13]	Mo—0.45 wt% C	Layer thickness—0.03 mm, hatch distance—0.1 mm, island scanning, zig-zag pattern, layer rotation −67°, layer shift—0.5 mm, energy input—0.66 J/mm Substrate plate temperature 800 °C	99.6 ± 0.2%	Bending strength—1180 ± 310 MPa Vickers hardness—343 ± 5 HV10
	Mo		97.7 ± 0.2%	Bending strength—267 ± 51 MPa Vickers hardness—208 ± 4 HV10
[14]	Mo	Line energy density—1142 J/m Scanning rotation—67° Spot size—75 µm	99.1%	
[15]	Mo	Laser power—100–350 W Scan speed—400–4000 mm/s Layer thickness—20–60 μm Hatch distance—70 μm Substrate temperature—150 °C Layer rotation—67°	>90%	
[17]	Mo	Laser power—200 W Layer thickness—0.02 mm Hatch distance—0.05 mm Scanning speeds—200–1000 mm/s.	NA	Flexural strength—329 MPa
[18]	Mo	Laser speed—314 (100–600) mm/s, build atm—3.3% H_2_/96.6% N2 (0–5% H_2_ in N_2_)	NA	Ultimate tensile stress—835 MPa, yield stress—760 MPa, ultimate tensile strain—0.017 mm/mm, final strain—0.01 mm/mm, Young’s modulus—77,313 N/m^2^.
[19]	Mo	Layer energy density—0.51 J/mm Laser power—200 W Velosity—400 mm/s Overlap rate—20%	99.1%	Hardness—260 HV Bending strength—280 ± 52 MPa

The reason for formation of pores and cracks can be different [19]. At lower energy density, the temperature of the molten pool is low, and the liquid phase volume is not enough to fill the voids. At higher energy density, Marangoni flow can drag gas, forming pores. The formation of the cracks is mainly explained by the fast solidification of the molten pool. Molybdenum has high thermal conductivity [20] which leads to a high cooling rate. The solidified part shrinks, creating stresses and strain, which forms cracks. It is proposed that the high oxidation sensitivity and low wettability of molybdenum also contributes to crack formation. The oxygen which accumulates in grain boundaries in the form of oxide has high evaporation pressure, resulting in increased spatter formation and an alteration of the Marangoni convection [21]. Crack formation can be inhibited by adding reinforcements [22], adding a support structure, suppressing the oxygen content [19], or by decreasing the thermal stresses by heating the powder bed [21].

The aim of this work is to manufacture molybdenum-based alloy on silicon carbide substrate using selective laser melting technology. The purpose of this system is to be able to prepare electrical circuits on SiC-based supports for the space industry. The idea is to develop technology to manufacture these multimaterial components in one-stage SLM. Silicon carbide containing 10 wt.% BN prepared by SLM technology [23] is proposed as substrate. Molybdenum containing 5 wt.% silicon was used as electrically conducting material.

## 2. Materials and Methods

Silicon carbide-based substrate manufacturing technology is described by Ghaltaghchyan et al. [23]. Then, the powder was eliminated from the chamber. The substrate was polished and Mo-5Si powder was placed.

The Mo-5Si powder feedstock was prepared using 95 wt.% molybdenum (Truer, Shanghai, China) (particle size 15–40 μm, purity ≥ 99.9% trace metals basis) and 5 wt.% silicon (Silgrain, Elkem ASA, Svelgen, Norway) particle size 10–30 μm). The powders were placed in a zirconia jar along with 10 mm zirconia balls. The powder-to-ball mass ratio was 1:2 by weight. The jar was rotated for 2 h at 50 rpm.

The selective laser melting process was performed using the Realizer GmbH SLM-50 system (DMG Mori, Bielefeld, Germany), with a 120 W continuous fiber laser (YAG: Nd^3+^) with a wavelength of 1.06 μm. Molybdenum −5 wt.% silicon (Mo-5Si) tracks with diameter of 100 μm and 400 μm and length of 15 mm were SLM-ed on SiC layer. To understand the possible reactions under SLM, solid samples of Mo-5Si were built with dimensions of 5 × 8.5 × 0.5 mm. Laser parameters included a power of 80 W, layer thickness of 30 μm, hatch distance of 0.06 mm, exposure time of 40 µs, and point distance of 20 µm. Continuous scanning was carried out with a 90-degree rotation between layers. Additionally, the silicon carbide baseplate was preheated up to 100 °C.

To assess porosity and microstructural features, rectangular samples were surface polished using a universal grinding and polishing device, the Qpol Go (Mammelzen, Germany). Microstructural characterization was performed by scanning electron microscope (SEM), (Zeiss, Evo 10, Carl Zeiss, Oberkochen, Germany) equipped with an EDS detector (Carl Zeiss, Oberkochen, Germany). Samples were coated with a 30 nm layer of gold to ensure enough conductivity. Phase characterization was performed by Mini Flex 600 X-ray diffractometer, Rigaku (Tokyo, Japan) (40 mA, 40 kV, Cu Kα radiation, λ = 0.1542 nm, step size of 0.02°).

The Vickers hardness of the samples was measured using the FALCON 600G2FA (INNOVATEST Europe BV, Maastricht, The Netherlands). A load of 1.0 kgf was applied for a dwell time of 10 s. Measurements were conducted at room temperature, with microhardness tests performed at different positions, to determine the mean value. At least 10 measurements have been performed.

Electrical resistance of the SLM-ed samples was measured by Cryomech (model No ST405, Cryomech, Syracuse, NY, USA) by a four-probe method. Current was applied and voltage was measured with nanovoltmeter (Keithley 2182A, Tektronix INC, Cleveland, OH, USA). The distance between the probes was s = 2.4 mm. The thickness of the samples was 0.75 mm, much thinner than the 40% of the distance between the probes; hence, no coefficient correction was needed. For each sample, 15 measurements were taken, 5 measurements for I = 0.5 mA, 5 measurements for I = 0.7 mA, 5 measurements for I = 1 mA. Mean resistance, resistivity, and standard deviations were calculated. The resistance then was calculated using the resistance Equation (1) for the sheet:

(1) $$R_{s}=\frac{\pi }{ln2}\times \frac{\Delta V}{I}$$

where R_s_ is the sheet resistance, ΔV is the change in voltage measured between the inner probes, and I is the current applied between the outer probes.

The resistivity equation reads (Equation (2))

(2) $$\rho =R_{S}d$$

where ρ is the resistivity and d is the thickness of the material.

## 3. Results and Discussion

**Figure 1:**
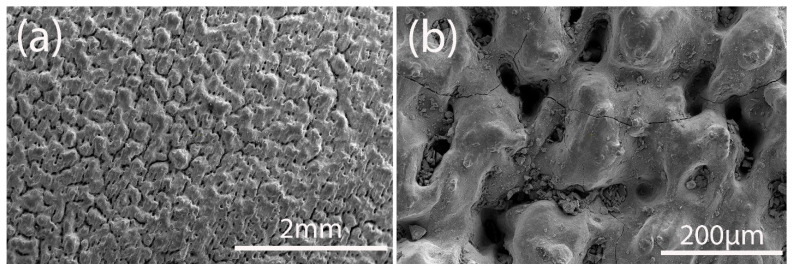
Microstructure of SiC-based substrate under (**a**) low and (**b**) high magnification.

Silicon carbide-based substrate was successfully manufactured. The grains are connected to each other with necks with clear orientation. This orientation corresponds to scanning orientation. The grains are a few times bigger than the initial powder particles size. The sintering happened through liquid phase formation under the laser, which regrouped the particles. The pores are distributed between the grains (Figure 1a). There are cracks on the SiC-based substrate, which can be caused by the fast heating and cooling rate of the laser sintering combined with high thermal conductivity of silicon carbide (Figure 1b).

**Figure 2:**
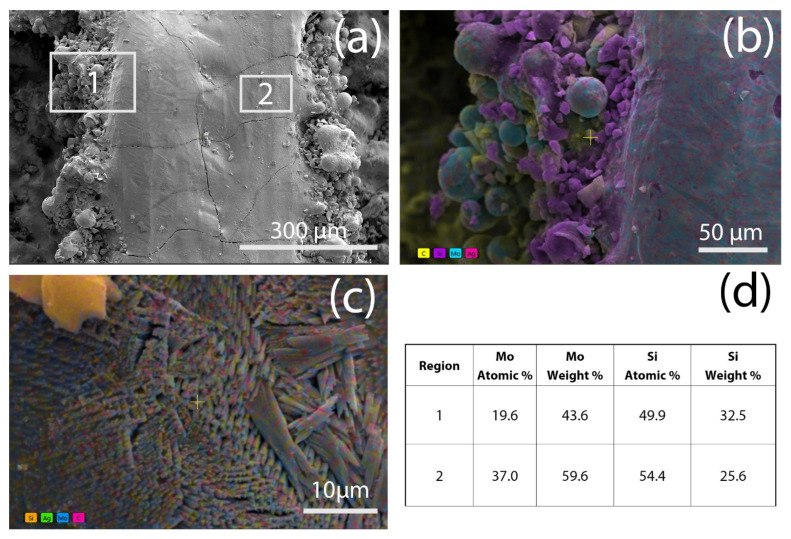
Microstructure of (**a**) Mo-5Si track, (**b**) high magnification image of Region 1, (**c**) high magnification image of Region 2, (**d**) EDS analyses of Regions 1 and 2.

The surface of the Mo layer is smoother (Figure 2a). There are cracks on the metal track, which is typical for SLM of molybdenum. The formation of cracks is due to high thermal conductivity, resulting in rapid solidification following the laser’s passage over the melting pool. The silicon was used because it was believed to react with molybdenum, generating energy [24]. The generated energy was proposed to decrease the colling rate and the thermal stresses. Moreover, the formed molybdenum silicides were proposed to prevent crack propagation. These cracks are likely superficial, and electrical conductivity remains. Functional evaluation will confirm the validity of the hypothesis.

According to the EDS study, molybdenum silicide-based spherical grains form on the edge of the molybdenum track (Figure 2b). In the middle of the track, submicron-sized fibers with Mo:Si atomic ratio of 37:54.4 are formed (Figure 2c,d), while in the corner of the tracks, the Mo:Si decreases. It should be noted the EDS analysis also reveals formation of carbon element.

**Figure 3:**
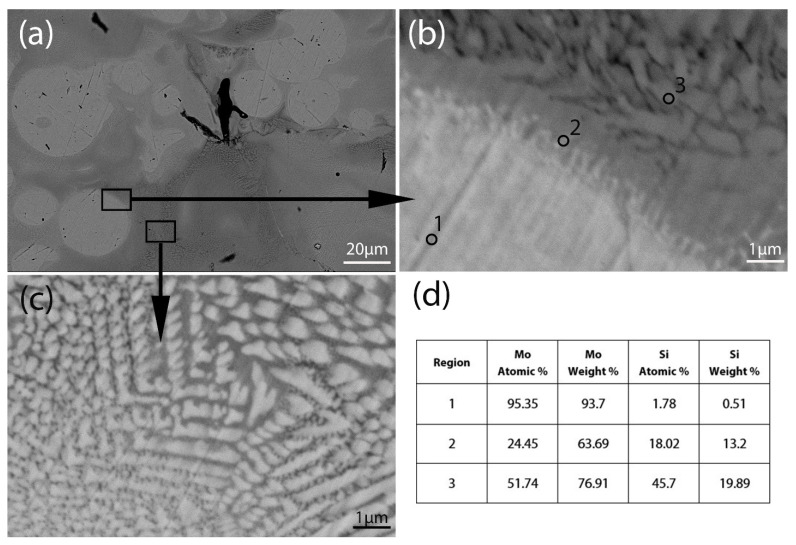
Microstructure of the polished cross section of Mo-5Si bulk sample: (**a**) low magnification, (**b**) high magnification image of the grain border, (**c**) high magnification image of the intergranular space, (**d**) EDS results of regions 1, 2, 3.

To understand the possible phases formed during Mo-Si laser melting, samples with 0.5 × 10.0 × 0.4 mm size were SLM-ed. The microstructure of the cross section of the sample is illustrated in Figure 3a. The EDS analysis shows that the grains are composed of mainly molybdenum (region 1), which is covered with a silicide layer (region 2) (Figure 3b). The EDS analysis shows that the layer contains a high amount of molybdenum, which probably corresponds to Mo_5_Si_3_ phase. Between molybdenum particles, there is a lumpy structure (Figure 3c). This matrix contains more molybdenum, according to EDS results (Figure 3d).

**Figure 4:**
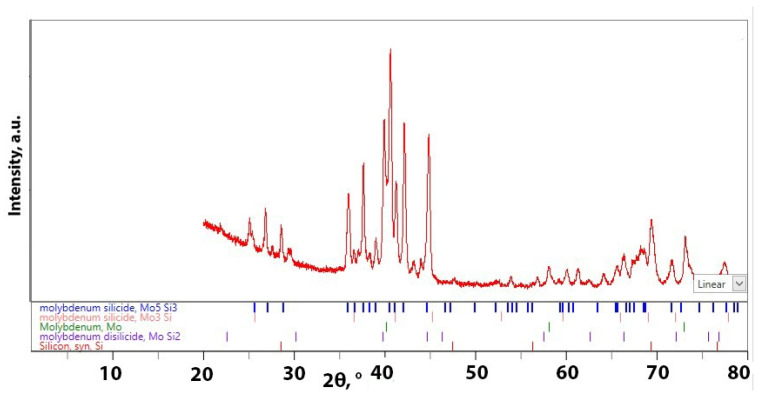
XRD of Mo-5Si sample.

The XRD analysis shows that the Mo_5_Si_3_ phase was primarily the main silicide formed during SLM (Figure 4). However, some MoSi_2_ and traces of Mo_3_Si were also formed. Semiquantitative analysis showed that ~55% of the weight fraction of the SLM-ed sample was Mo_5_Si_3_. Mo_3_Si and molybdenum were the subsequent larger quantity of phases. It can be concluded that the bright lumpy structure formed between molybdenum particles illustrated in Figure 3c is Mo_5_Si_3_. Some authors revealed that Mo_5_Si_3_ is generated due to solid–solid interaction during the preheating period [25]. MoSi_2_ phase forms just after silicon melts [26]. The Mo and MoSi_2_ dissolves in Si melt, and when silicon melt becomes saturated or supersaturated, Mo_5_Si_3_ and Mo_3_Si precipitate. It is revealed that Mo_5_Si_3_ and Mo_3_Si are formed in the absence of silicon melt. It is proposed that Mo_5_Si_3_ formed during the colling staged at SLM process. Mo_5_Si_3_ has higher formation enthalpy than Mo_3_Si; hence, formation of Mo_5_Si_3_ phase is more likely [27]. The presence of Mo_3_Si could be caused by the diffusion interaction of Mo with Mo_5_Si_3_, which takes a longer time.

### Mechanical Properties

Vickers hardness of the samples was measured by applying 1.0 kgf load. There are radial microcracks generated from one or two sides of the indentation. As the microcracks are not linear and have different length, the fracture toughness was not measured. The value of Vickers hardness of the selective laser melted Mo-5Si is 7.6 ± 1.0 GPa, which is a few times higher than referred before [12,19].

**Figure 5:**
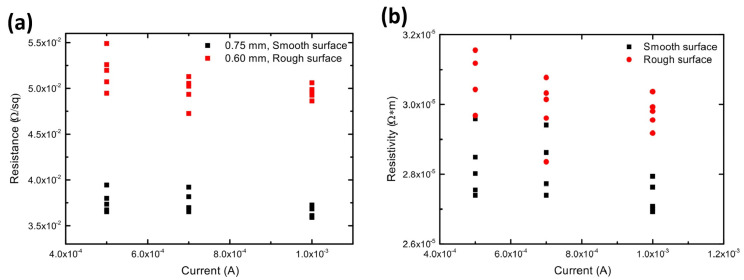
Four-probe measurement results: (**a**) resistance versus current and (**b**) resistivity versus current.

Electrical resistivity of the samples before and after polishing was measured using a four-probe method by applying low and high current. The resistance and resistivity dependence on current is illustrated on Figure 5. There is slightly bigger deviation (<3.6%) when a small current is applied, while the results are more precise for high current applied: the deviation then is <1.7%. Similar to resistance statistical analyses, there is slightly bigger deviation (<3.6%), when a small current is applied, while the results are more precise for high current applied: deviation then is <1.7%.

It would be expected that resistivity was the same for different samples; however, roughness could lead to increased resistivity. The value of the resistance and resistivity of the samples with both rough and smooth surfaces is concluded in Table 2. Surface roughness leads to increased resistivity; meanwhile, the same material may have lower resistivity if the surface is polished. This should be considered when designing the further manufacturing process.

## 4. Conclusions

Selective laser melting was successfully applied to obtain multilayer metal–ceramic-based composites in one stage.

The silicon carbide layer manufactured by SLM has a glassy matrix that has aided its sintering and a residual intergranular porosity.

Molybdenum mixed with 5 wt.% silicon was SLM-ed on the SiC layer, showing a smooth surface.

Molybdenum reacted with silicon during the laser melting process. Molybdenum silicides were formed.

The electrical conductivity results confirm the hypothesis and the production of Mo-based conductive wires obtained using SLM.

## Figures and Tables

**Table 2:** Descriptive statistics of the resistance and resistivity at high current.

**Resistance**			
**Data**	**N Total**	**Mean/Ω**	**Standard Deviation**
smooth surface	5	0.03642	5.94953 × 10^−4^
rough surface	5	0.04961	7.3647 × 10^−4^
**Resistivity**			
**Data**	**N Total**	**Mean/Ω·m**	**Standard Deviation**
smooth surface	5	2.73167 × 10^−5^	4.46215 × 10^−5^
rough surface	5	2.97681 × 10^−5^	4.41883 × 10^−5^

## Data Availability

The original contributions presented in this study are included in the article. Further inquiries can be directed to the corresponding author.
